# A patient with bacteraemia and possible endocarditis caused by a recently-discovered genomospecies of *Capnocytophaga: Capnocytophaga genomospecies AHN8471*: a case report

**DOI:** 10.1186/1752-1947-2-369

**Published:** 2008-12-04

**Authors:** Jonathan M Mills, Emma Lofthouse, Phil Roberts, Johannis A Karas

**Affiliations:** 1 Department of Microbiology, Hinchingbrooke Heath Care NHS Trust, Huntingdon, PE29 6NT, UK; 2 Department of Medicine, Hinchingbrooke Heath Care NHS Trust, Huntingdon, PE29 6NT, UK

## Abstract

**Introduction:**

*Capnocytophaga *are a genus of bacteria that have been found to be the causative organisms in a range of infections, including serious conditions such as bacteraemia, endocarditis and meningitis. This has been especially true amongst those with serious comorbidities and the immunocompromised populations. Although several species are known to cause human disease, historically, laboratories have often not identified isolates to species level due to the unreliable, laborious techniques needed. With the advent of Polymerase Chain Reaction-Restriction Fragment Length Polymorphism Analysis, identification to species level is now frequently possible and desirable, as it may provide clues as to the source of infection and its treatment.

**Case presentation:**

Here we describe a case of bacteraemia and possible endocarditis in a 64-year-old white British man caused by a newly identified genomospecies of *Capnocytophaga *in a patient subsequently diagnosed with metastatic adenocarcinoma of the oesophagus. The source of the bacteraemia was presumed to be from the patient's own oral flora.

**Conclusion:**

Our case further confirms the potential for *Capnocytophaga *to cause systemic infections, highlights the availability and need for identification of isolates to species level and re-emphasises the difficulty in diagnosing *Capnocytophaga *infections due to their slow growth in the laboratory.

## Introduction

The members of the genus *Capnocytophaga *are a group of capnophilic, facultatively anaerobic, Gram-negative bacilli that inhabit the oropharyngeal cavity of both humans and animals [1]. Their association with human periodontitis has been well documented [2]. More seriously, they have been regularly reported to be the causative agents in unusual cases of septicaemia and endocarditis, with immunosuppressed, asplenic or alcoholic patients at particular risk [3]. They have also been more rarely implicated in a wide range of infections including meningitis, haemolytic uraemic syndrome, spontaneous bacterial peritonitis, septic arthritis, and hepatic and cerebral abscesses [4-9]. Dog or cat bites have been shown to be a particular risk factor for transmission of *Capnocytophaga canimorsus*, which has the potential to cause severe sepsis in humans. In the past, many authors have simply reported the infectious agent as *Capnocytophaga *sp., due to the difficulty of laboratory identification of individual species by morphological or biochemical means alone [5]. Molecular techniques now allow accurate speciation, with the use of Polymerase Chain Reaction-Restriction Fragment Length Polymorphism Analysis (PCR-RFLP). Identification to species level is desirable as it may indicate the likely source of the infection and guide subsequent investigation or treatment.

Here we describe a case of bacteraemia caused by a recently discovered genotype of *Capnocytophaga*, *Capnocytophaga genomospecies AHN8471 *(GenBank accession number DQ009622).

## Case presentation

A 64-year-old white British man was referred urgently to hospital with a 2-month history of dysphagia, anorexia, nausea, significant weight loss (greater than 15 kg), general fatigue and insomnia. He also reported a 3-day history of a husky, weak, hoarse voice. He was a lifelong heavy smoker (50 pack years) and had formerly had an excessive alcohol intake. On examination, he was apyrexial with no lymphadenopathy, but was noted to have clubbing, splinter haemorrhages, gynaecomastia and spider naevi. Cardiovascular and respiratory examinations were otherwise unremarkable, but examination of the abdomen showed the liver edge to be just palpable. Admission blood tests showed: haemoglobin 12.9 g/dL, white cell count 6.2 × 10^9^/L, alanine aminotransferase 49 U/L, alkaline phosphatase 234 U/L, albumin 26 g/L, gamma-glutamyl-transferase 192 U/L, C-reactive protein 108 mg/L. Urinalysis showed evidence of microscopic haematuria, and a chest radiograph revealed non-specific bilateral reticulonodular shadowing. A clinical diagnosis of endocarditis was made, on a background of possible oesophageal malignancy. Two sets of blood cultures were obtained on the day of admission and a further four sets over the subsequent 4 days, during which time the patient had a low-grade pyrexia.

Computed tomography scanning revealed thickening of the distal oesophagus with hilar lymphadenopathy, hepatic lesions consistent with metastases, a mass in the left adrenal gland, several areas of renal infarction bilaterally, but no cerebral metastatic deposits. Barium swallow confirmed a long shouldered stricture in the mid oesophagus in keeping with an oesophageal carcinoma. Trans-thoracic echocardiography revealed no abnormalities.

The first set of blood cultures taken on the day of admission became positive after 7 days incubation. Gram-negative rods were isolated from both aerobic and anaerobic bottles after sub-culture on blood agar. The isolate was catalase and oxidase negative and susceptible to ampicillin and cefotaxime. The isolate could not be identified further and was sent to the Health Protection Agency Centre for Infections Laboratory, Colindale, London. It was identified as *Capnocytophaga genomospecies AHN 8471 *by 16S rRNA PCR-Restriction Fragment Length Polymorphism as previously described [10]. A second set of blood cultures taken on the day following admission were found to be positive in both bottles after less than 24 hours incubation. Gram-positive cocci were isolated and subsequently identified as *Streptococcus mitis*. Four further sets of blood cultures obtained over the subsequent 4 days were negative upon incubation. Given the clinical features of endocarditis, the patient was commenced on benzylpenicillin 2.4 g 6 hourly by intravenous infusion plus gentamicin 80 mg 8 hourly by intravenous infusion on day 6 of admission. It was while he was on this treatment that the identification of the original blood culture isolates as *Capnocytophaga *spp. became known.

The patient went on to have an oesophagogastroduodenoscopy (OGD) that enabled visualisation of the malignant lesion, biopsies of which revealed squamous cell carcinoma. Subsequently, he had endoscopic laser ablation therapy of the tumour. Although he was being treated for endocarditis, and would have thus warranted more prolonged intravenous antibiotic therapy, his malignancy was at such an advanced stage that he was discharged home to be with his family. He completed a further 5 days of intravenous ceftriaxone 2 g daily after discharge, administered at home. The source of infection with *Capnocytophaga *in this patient is likely to have been from his own mouth flora and the clinical suspicion of endocarditis could not be confirmed.

Over the next few weeks, he underwent palliative oesophageal stenting, but there was felt to be no role for chemotherapy or further intervention. He deteriorated and died 2 months after the initial presentation.

## Conclusion

Our case shows again the potential for *Capnocytophaga *to cause bacteraemia and systemic illness in humans. Patients with serious comorbidities are particularly at risk. The case also highlights the fact that *Capnocytophaga *typically exhibit slow growth, often requiring several days of incubation. The possibility of *Capnocytophaga *infection should thus be borne in mind in cases of so-called 'culture-negative' endocarditis, especially if the clinical history is suggestive. The ability to identify the organism to species level may be useful in diagnosis or management. *Capnocytophaga *have variable susceptibility and can be susceptible to penicillins, extended spectrum cephalosporins and quinolones.

This is the first report in the literature of bacteraemia caused by this genotype of *Capnocytophaga, genomospecies AHN8471*; indeed, this genotype has only recently been described, as a member of the normal oral flora of healthy children [11].

## Consent

Written informed consent was obtained from the patient's wife for publication of this case report and any accompanying images. A copy of the written consent is available for review by the Editor-in-Chief of this journal.

## Competing interests

The authors declare that they have no competing interests.

## Authors' contributions

All authors carried out the clinical work, and read and approved the final manuscript.
